# Evaluation of One- and Two-Box Models as Particle Exposure Prediction Tools at Industrial Scale

**DOI:** 10.3390/toxics9090201

**Published:** 2021-08-29

**Authors:** Carla Ribalta, Ana López-Lilao, Ana Sofia Fonseca, Alexander Christian Østerskov Jensen, Keld Alstrup Jensen, Eliseo Monfort, Mar Viana

**Affiliations:** 1 The National Research Center for Work Environment (NRCWE), DK-2100 Copenhagen, Denmark; agf@nfa.dk (A.S.F.); alj@nfa.dk (A.C.Ø.J.); kaj@nfa.dk (K.A.J.); 2 Institute of Ceramic Technology (ITC)-AICE, Campus Universitario Riu Sec, Universitat Jaume I, 12006 Castellón, Spain; ana.lopez@itc.uji.es (A.L.-L.); eliseo.monfort@itc.uji.es (E.M.); 3 Institute of Environmental Assessment and Water Research (IDAEA-CSIC), 08034 Barcelona, Spain; mar.viana@idaea.csic.es

**Keywords:** mass-balance models, occupational exposure, dustiness index, handling energy factor, indoor aerosol modelling, emission rate, exposure assessment

## Abstract

One- and two-box models have been pointed out as useful tools for modelling indoor particle exposure. However, model performance still needs further testing if they are to be implemented as trustworthy tools for exposure assessment. The objective of this work is to evaluate the performance, applicability and reproducibility of one- and two-box models on real-world industrial scenarios. A study on filling of seven materials in three filling lines with different levels of energy and mitigation strategies was used. Inhalable and respirable mass concentrations were calculated with one- and two-box models. The continuous drop and rotating drum methods were used for emission rate calculation, and ranges from a one-at-a-time methodology were applied for local exhaust ventilation efficiency and inter-zonal air flows. When using both dustiness methods, large differences were observed for modelled inhalable concentrations but not for respirable, which showed the importance to study the linkage between dustiness and processes. Higher model accuracy (ratio modelled vs. measured concentrations 0.5–5) was obtained for the two- (87%) than the one-box model (53%). Large effects on modelled concentrations were seen when local exhausts ventilation and inter-zonal variations where parametrized in the models. However, a certain degree of variation (10–20%) seems acceptable, as similar conclusions are reached.

## 1. Introduction

In occupational hygiene, there is a need to assess worker exposure for a large variety of microenvironments and materials in order to guarantee workers safety. In this regard, the REACH regulation [1] requires estimation of human exposure by all relevant routes to determine the appropriate risk management measures and prevent excessive exposure. The EN 689:2018+AC:2019 [2] standard provides indications in this sense and recommends a basic exposure characterization in order to decide if personal exposure measurements are needed, which can be obtained by using adequate exposure models as shown in Koivisto et al. [3]. Mass-balance models have been proposed by several authors as useful tools for indoor particle exposure prediction [4,5,6,7]. Among many mathematical models, the one- and two-box models are some of the most widely used and have been shown to have relatively good performance [8,9] despite having low input and mathematical requirements. Performance of one- and two-box models has been evaluated by several authors mainly under highly controlled environments [8,10,11], and in specific real-world industrial scenarios [9,12,13]. However, in order to build trust in the use of quantitative exposure prediction models for exposure assessment and management, extensive testing against real-world cases are needed to test model performance and to understand the uncertainties related to critical parameters such as source characterization, local controls and air mixing, which are not yet fully parametrized and understood [14,15]. These determinants of exposure are often challenging to estimate in real-world scenarios [9,16,17,18]. In addition, information on contextual information may be known with different levels of detail depending on the actual use of the model.

For source characterization and emission rates determination when dealing with powdered materials, the most used input parameter is the dustiness index (DI), which is a measure of a material’s tendency to generate airborne dust during mechanical or aerodynamic stimulus. In some tools, dustiness can be introduced based on appearance, but it can be relatively easily determined under laboratory conditions following standardized methodologies (EN 15051; EN 17199). In particular, the rotating drum (RD), and small rotating drum methods were developed to simulate processes that involve repeated dropping, pouring, and agitation of bulk powder, granulates materials, and the like, while the continuous drop (CD) method simulates powder pouring in a continuous feed. On the other hand, the vortex shaker is intended to simulate worst-case scenarios of handling processes where a high-frequency vibration and high energy input or high activation energy is applied to the powder or bulk material. Therefore, the use of different dustiness methods will result in considerable differences in the dustiness values. If such dustiness data are used directly, the emission rates or exposure scaling will potentially be different. Consequently, in quantitative tools, the process-specific emission rate is typically estimated considering the mass handled per fraction of time during the process under study and a so-called handling (energy) factor (H) to adjust the method-specific DI to the process scenario. Several risk and exposure assessment tools, e.g., Stoffenmanager, ART or ECETOC TRA use the DI as input parameter, which is later scaled to the specific process [19,20]. H links by definition the effective mechanical energy applied during a specific process with the energy used during the dustiness test [21] and has been traditionally defined to range from zero to one [14,22]. However, this may not be true in cases where the effective dispersion energy applied during the process is higher than the applied during the dustiness test. In this regard, different dustiness test methods give different results and therefore the quantitative scaling of the H factor for different work scenarios will vary. However, this is not yet well fully characterized. In this regard, current work is ongoing under H2020 EU projects (Gov4Nano and NanoHarmony) aiming to develop a guidance document on how dustiness data can be used for worker exposure assessment. Up to date, several studies have been published regarding dustiness parametrization and its use on modelling although further research is still required [15,23,24].

In addition, local controls, including all those actions taken to prevent the dispersion of the aerosolized particles or to remove particles from air, e.g., enclosures or local exhaust ventilation (LEV) systems, need to be included in the equations as an estimated or calculated factor of reduction. Reduction percentages due to local controls can be relatively easily calculated [25,26], but especially in real-world scenarios they can be complex to estimate. Thus, some authors Fransman et al. [27], revised by Goede et al. [28] have presented an integrated risk management measure library, which contains efficacy values for different local controls, which can be used when specific data are missing. These values are now publicly available online through ECEL v3.0 (https://diamonds.tno.nl/info/87, accessed on 14 June 2021). The use of literature reported values for indoor modelling has already been conducted by some authors and seems to be promising in order to tackle the lack of some parameters [7,13].

Finally, general air changes per hour (ACH) and indoor air flows are known to be key parameters that can be difficult to accurately estimate when modelling real-world industrial scenarios. Theoretically, air flows should be relatively easy to obtain as only air speed velocities are needed. However, too often several practical problems are encountered. On one hand, important differences are frequently seen between using air speeds for ACH calculation or tracer gas measurements, the latter one being considered more appropriate for modelling [29]. Additionally, indoor air flows can be difficult to measure due to the complex interactions between room air currents, LEV systems, local controls or temperature [26] as well as contributions from activity and moving parts. Thus, in many cases indoor air flows will need to be assumed from literature or dispersion tests in the facility if this option is available.

Due to the lack of full model parametrization and characterization, more studies dealing with real-world modelling needs to be conducted in order to better understand the variation in parameters that control exposure and how these variations can be covered in the models. Currently, studies on models evaluation for the same process but with changing parameters (e.g., type of material, energy applied or local controls) are very limited, constraining the assessment of model performance, applicability and reproducibility for real-scenarios.

The aim of the present study is to evaluate the performance of the one- and two-box models under real-world industrial scenarios. In this context, data from a previous study [30] where seven different ceramic raw materials were packed in three filling lines with different levels of energy and mitigation strategies applied was used. Modelling was conducted by using the same criteria for characterization in order to test model performance, applicability and reproducibility. In addition, a one-at-a-time (OAT) methodology was applied to conduct a sensitivity analysis to provide insights on the effects of parametrization on model performance.

## 2. Materials and Methods

### 2.1. Materials and Work Environment

**Figure 1:**
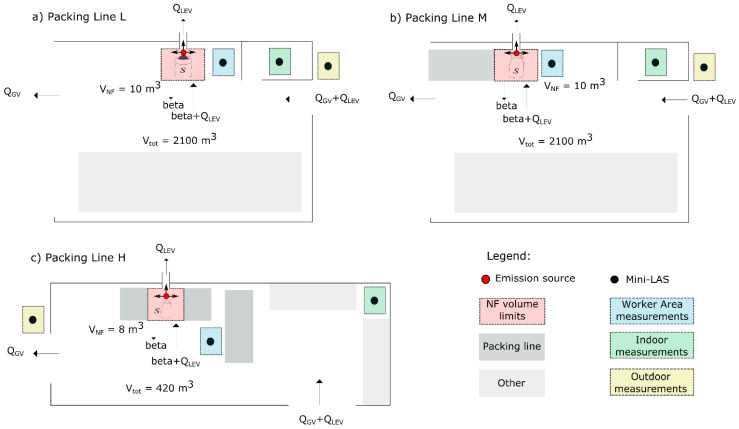
Modelling layout for Industrial Setting #1 containing filling lines L (**a**) and M (**b**), and for Industrial Setting #2 containing filling line H (**c**).

Filling of 7 ceramic materials (2 clays (>90% clay; CAS: 999999-99-4), 2 feldspars (>90% feldspar; CAS: 68476-25-5 and respirable crystalline silica between 1 and 10%), 2 kaolins (>90% kaolinite; CAS: 1332-58-7) and 1 quartz (>95% quartz; CAS: 014808-60-7)) in 3 different filling lines with different energy levels and mitigation strategies applied was monitored between the 14th and 28th of February 2018 at 2 industrial settings, named as #1 and #2, in the vicinity of Valencia, Spain (Figure 1 and Supplementary Table S1). Hereafter, filling lines L, M and H refer to “low”, “medium” and “high” levels of mitigation measures implemented. Materials characteristics are shown in Supplementary Section S1 and Table S1. More detailed information on materials and exposure results is reported and fully described in Ribalta et al. [30].

Industrial Plant #1: Filling lines L and M were located in Plant #1, with a total volume of 2100 m^3^ (Figure 1a,b). Filling of big bags (1200 kg) was carried out through a cylindrical opening. Both filling lines were not enclosed but were equipped with a LEV, with a theoretical flow rate of 18,000 m^3^ h^−1^ (Q_LEV_), and a subsequent bag filter. Filling line L had not a seal system for attaching the bags to the feed funnel whereas a partially closed seal system was in place at filling line M to reduce release of airborne dust. In both cases, the feed funnel was placed inside the bags, with an open-air drop of 0. The maximum material drop height inside the bags was 1.3 m from the feed to the bottom of the bags in both filling lines. Filling lines L and M were not operated at the same time but diesel-powered forklifts were used to move the filled bags to the storage area and occasionally other activities were performed in the plant.

Industrial Plant #2: with a total volume of 420 m^3^, contained filling line H where filling of small bags (20–25 kg) was carried out through a lateral cylindrical opening (Figure 1c). The maximum material drop height was 0.6 m. The filling line was not enclosed but during filling the bags were sealed to the feed funnel equipped with a LEV system (Q_LEV_) (flow rate of 2400 m^3^ h^−1^) with a subsequent bag filter.

In addition to LEV, both plants were naturally ventilated with air coming from outdoors via open doors (Q_GV_). Indoor air velocities were measured, and used to calculate flows (m^3^ min^−1^) and the total general ACH for each day (see Supplementary Table S1).

### 2.2. Aerosol Measurements

Particle number and mass concentrations were monitored in real time by using mobility and optical particle sizers, aerosol photometers, diffusion chargers and a condensation particle counter. In this work, only results from the Mini Laser Aerosol Spectrometer (Mini-LAS 11R, Grimm Aerosol Technik, Ainring, Germany) to measure particle mass concentration from 0.25 to 32 µm in 31 channels with a 1-min time resolution and 1.2 L min^−1^ sample flow rate were used. Description of all instrumentation used can be found in Ribalta et al. [30], and the uncertainties of the portable instruments are reported in Viana et al. [31] and Fonseca et al. [32].

Monitoring was conducted in the worker area (WA), indoors, and outdoors simultaneously with the Mini-LAS. The instrument in the WA was placed on a portable table at approximately 1 m height (instrument inlets being at 1.5 m above the ground level) and at around 2.5 m from the centre of emission source and 1.5 m from the limits of the near-field (NF). All instruments were synchronized prior to the measurements and intercompared.

Air speeds inside the plant (at WA) were experimentally measured with a Weather Transmitter WXT536, WXT530 Series, Vaisala, Helsinki, Finland.

### 2.3. Dustiness

Material DI was assessed by using the CD and the RD standard methods (EN 15051:2013). Dustiness index results (mg kg^−^^1^) as well as categorical ranking of the powders according to EN 15051 classification are presented in Table 1 and described in detail in the Supplementary Section S2 and Supplementary Table S2.

The CD device, made of stainless steel, consists of a cylindrical pipe through which air circulates in an upward direction with a volume flow rate of 53 L min^−^^1^ [33]. Sampling heads for inhalable (designed by Institut für Gefahrstoff-Forschung—IGF) (W_I_) and respirable (FSP-2, BGIA) (W_R_) fractions are located slightly above the discharge position of the material. Samples for gravimetric measurements of inhalable and respirable fractions were collected on cellulose thimbles, single thickness, 10 × 50 mm, and PVC filters of 37 mm Ø and 5 µm of porosity. Total material drop height during the test was approximately 1.2 m.

The RD test as described in the standard EN 15051 (Part 2), consists of introducing a known volume of material (35 mL) inside a rotating drum (Ø 30 cm = approximately total material drop height during test) which rotates at 4 revolutions per minute. The dust generated inside the drum is collected onto a three-body sampling system, in which the emitted dust cloud is drawn by the air current generated by a vacuum pump at a flow rate of 38 L min^−^^1^. The dust sampling system consists of two sections of selective foam per particle size (one metal coated PE foam of 20 ppi and one metal coated PE foam 80 ppi) followed by a glass fibre filter, to gravimetrically analyse inhalable (W_I_), thoracic (W_T_) (data not shown) and respirable (W_R_) fractions.

### 2.4. One- and Two-Box Models

Exposure modelling was performed by using a one-box [34] and a two-box model [26]. The models assume that (1) particles are fully mixed at all times; (2) mass is created by a source inside the plant (NF in two-box model) and; (3) particle losses are only due to natural and mechanical (LEV) ventilations. The models were used to calculate the inhalable and respirable fractions. Particle losses by sedimentation and coagulation were not considered.

#### 2.4.1. Emission Source Characterization and Parametrization

The emission (S(t)) from the filling process is described based on the DI as:

(1) $$S\left(t\right)=\;\mathrm{DI}\cdot H\cdot \frac{\mathrm{dM}\left(t\right)}{\mathrm{dt}}\cdot LC_{bag}\cdot LC_{LEV}$$

where DI is the inhalable or respirable dustiness index (CD or RD) of the material expressed in mg kg^−^^1^ (Table 1), H is the handling energy factor for the process, dM(t)/dt (kg min^−^^1^) is the mass flow of the material (Table 1), LC_bag_ is the local control reduction factor due to the presence of the bag and attachment to the feed funnel, and LC_LEV_ is the reduction due to the LEV effect. As efficiency reductions due to the enclosure of the bags could not be experimentally determined, literature values were used. Enclosures have been reported to reduce emissions from 10% up to more than 90% [25,26,27]. Additionally, several search combinations were made in the Exposure Control Efficacy Library (ECEL v3.0) (data are shown in Supplementary Section S3) and results were used to select the different reduction efficacies applied in the modelling. For bagging and pouring processes “containment without ventilation” had a reported efficacy of 30–85% in the ECEL library (Supplementary Figures S1 and S2). On the other hand, for general processes “low and medium level containment” had reported efficacies mainly between 35–75% (with median approximately at 65%) and 30–100% (with median approximately at 95%), respectively (Supplementary Figures S3 and S4). Based on these values, the reduction in particle emissions due to the effect of the bag (LC_bag_) were chosen as 70, 80 and 90% reduction for filling line L, M and H, respectively (Table 1). These reduction percentages were introduced in Equation (1) as the LC_bag_ parameter. Similarly, for the LEV effect, no experimentally determined reduction values could be obtained. Thus, several reduction efficiencies were tested by using the OAT method in order to determine the impact on the model output (see Section 2.5). For bagging and pouring processes, “fixed capturing hoods” have reported efficacies between 50–90% in the ECEL library (see Supplementary Figures S5 and S6). Therefore, reduction values tested in the modelling were 50, 70, 80 and 90%. There are several standardized DI methods available, which intend to resemble different processes and activities, and thus provide different DI values. For modelling, it is advised to use the method, which most closely resembles the process under study. However, good dustiness/exposure correlations have been found during pouring of powders [25] when using both the CD and the RD dustiness methods. Thus, effects on modelling performance when using the CD and the RD, was studied. The handling energy factor was assumed to be 1 for filling in lines L and M, where bags of 1200 kg where packed, and 0.5 for filling line H, where small bags of 25 kg were packed (Table 1).

#### 2.4.2. One-Box Model

In the one-box model, the mass balance concentration inside the model volume is described as a function of time:

(2) $$V\;\frac{\mathrm{dC}}{d_{t}}=S+\;Q\cdot C_{0}-Q\cdot C$$

(3) $$Q=\left(Q_{GV}+Q_{LEV}\right)=ACH\cdot V$$

where S (mass or particle number min^−^^1^) is the emission source, Q (m^3^ min^−^^1^) is the total air flow including air flow due to general ventilation (Q_GV_) and LEV (Q_LEV_), ACH (h^−^^1^) is the air changes per h, V (m^3^) is the box volume, C is the (inside the box and outgoing) concentration and C_0_ is the initial and incoming concentration.

#### 2.4.3. Two-Box Model

In the two-box model, mass balance concentration inside the model volume (NF and far-field (FF) volume) is described as a function of time:

− Mass balance in the NF:

(4) $$V_{NF}\;\frac{dC_{NF}}{d_{t}}=S+\beta_{i}\cdot C_{\mathrm{FF}}-\beta_{i}\cdot C_{NF}$$

− Mass balance in the FF:

(5) $$V_{FF}\;\frac{dC_{FF}}{d_{t}}=\left(Q_{GV}+\;Q_{LEV}\right)\cdot C_{0}+\beta \cdot C_{\mathrm{NF}}-\beta_{i}\cdot C_{FF}-\;Q_{GV}\cdot C_{FF}$$

(6) $$Q=ACH\cdot \left(V_{NF}+V_{FF}\right)$$

where S (mg min^−^^1^) is the emission source in the NF, C_NF_ and C_FF_ are NF and FF concentrations, V_NF_ and V_FF_ (m^3^) is the volume in NF and FF, β is the air flow between NF and FF (m^3^ min^−^^1^), and β_i_ is the air flow between NF and FF including (Q_LEV_) air flow due to LEV (m^3^ min^−^^1^).

### 2.5. Model Parametrization and Evaluation

The use of the OAT methodology, with lower and upper parameter boundaries of a most likely, a min and max value, is proposed by several authors to analyse model performance [9,26]. This provides a range of predicted concentrations and allows for identification of model variables that contribute the most to output variability and uncertainty, as well as information on which parameters are the most crucial. In this work, the OAT approach was applied to the LEV reduction effect (LC_LEV_) and the inter-zonal (NF-FF) flow rate, β.

Measuring β can be problematic given the complex interactions between general room air currents and air-flow created by a.o. the local controls or the warm bodies [26]. When local controls are involved, as in this study, the flow rate entering the NF (β_i_) consists of both β and Q_LEV_, complicating the estimation of the existing β using air velocity measurements. Literature reported β values for several indoor environments range between 0.24–30 m^3^ min^−^^1^ [7,8,10,18,35], with average values around 5 m^3^ min^−^^1^. Therefore, 5 m^3^ min^−^^1^ was considered most likely β value and 0.25, 0.5, 1, 2.5, and 10 m^3^ min^−^^1^ were tested. Flow values from FF to NF due to LEV effect (Q_LEV_) were fixed at 10 and 5 m^3^ min^−^^1^ for filling lines L, M and H, respectively.

In occupational settings, discrimination of process-specific airborne particles from background concentrations has been pointed out as a key step. Therefore, when modelling a real-world scenario this must be taken into account. In Ribalta et al. [13], one- and two-box model accuracy and performance were seen to improve when including background concentrations. Therefore, in this study, the inclusion of background concentrations on modelling performance was also studied.

Model performance was evaluated according to the benchmark for the ratio modelled/measured concentrations of 0.5–2 reported by Jayjock et al. [9] which has been used by several authors [10,13,14,36]. The following nomenclature was used in this study (i) underestimation (ratio < 0.5), (ii) accurate estimation (ratio 0.5–2), (iii) slight overestimation (ratio 2–5) and (iv) high overestimation (ratio > 5). In addition, the criteria for models assessment proposed in Fransman et al. [37], which has been used to assess several exposure assessment tools, was also considered in order to evaluate models performance. Therefore, R^2^ and Spearman correlation coefficients were calculated (criteria; >0.6) as well as the percentage of measured values exceeding modelled values (criteria; <10%). Moreover, the descriptive statistical mean absolute error (MAE) was calculated.

## 3. Results and Discussion

The WA stationary monitored exposure concentrations, reported in Table 2 and described in Supplementary Section S4, were modelled by using the one- and two-box models, and an OAT analysis was conducted for two modelling input parameters (Q_LEV_ and β). Monitored WA measurements were compared to one-box and two-box FF model results as monitoring instruments were placed at 2 to 2.5 m from the emission source, therefore outside of the applied model limits of the NF. Modelling performance was assessed by considering dustiness method used as input for emission source, mass fraction modelled (inhalable and respirable) and type of model (one- and two-box). In addition, the effect of background concentrations, LEV efficacy reduction and β value were studied. Modelled concentrations and ratios of modelled/measured concentrations are given in Table 2. In addition, linear regression and Spearman correlation coefficient, as well as the statistical descriptors difference, absolute difference and mean absolute error (MAE) are provided in Supplementary Section S5, Tables S3 and S4, and Figures S7 and S8, respectively.

### 3.1. Dustiness Method and Modelled Exposure to Inhalable and Respirable Dust

The effect of one- and two-box models performance when using the CD or RD DI as input parameter was studied. The EN15051 dustiness test methods provide inhalable and respirable mass fraction, which are both regulated and thus of interest for the occupational hygiene community. Therefore, when studying model performance both mass fractions were considered.

**Figure 2:**
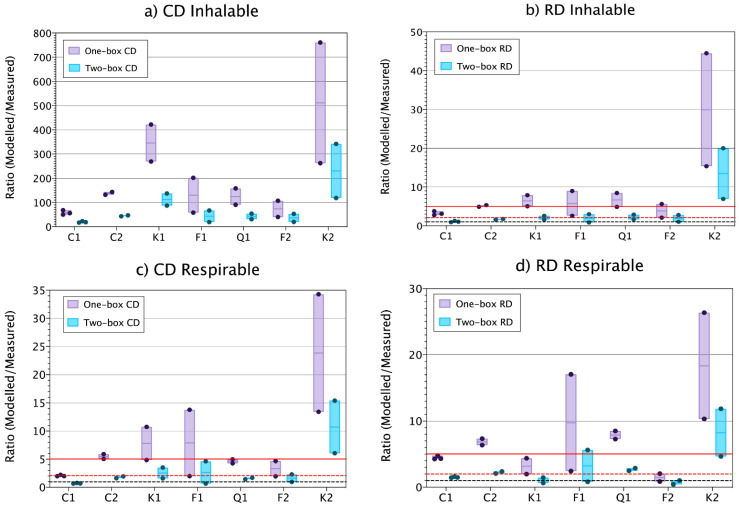
Vertical boxplot showing all materials one- (room) and two-box (FF) ratio modelled/measured (**a**) inhalable concentrations using CD DI as input, (**b**) using RD DI as input for the one-box model, (**c**) using CD DI as input for the two-box model, and (**d**) using RD DI as input for the two-box model. Ratios 1, 2 and 5 are marked as reference (red solid line, red dashed line and black dashed lines, respectively). The solid coloured line within the box indicates the median value, and the limits of the box indicate min and max values. Coloured dots represent the individual replicate values. Modelling parameters: LEV and β fixed at 70% and 5 m^3^ min^−1^, respectively.

Results show that inhalable mass fractions modelled with the one- (room) and two-box (FF) models were highly overestimated, with ratios > 15 in all cases, when using the CD DI. Conversely, when using RD DI, monitored inhalable mass concentrations were predicted with ratios < 5 on 53% and 87% of the cases for the one- and the two-box model, respectively (Figure 2a,b and Table 2). Thus, more accurate modelling results were obtained for inhalable mass fraction using the RD DI than the CD DI with the specific modelling settings selected. This was an unexpected result, as the CD approach is considered to resemble more closely the filling process. Hence, the result seems to go against the immediate logic to use the DI that most closely resembles the process under study. However, whereas large differences were obtained for the modelled inhalable fraction when using different DIs differences were smaller when modelling respirable mass. Using CD DI, 27 and 67% of the cases were accurately estimated (ratios 0.5–2) with the one- and two-box model, respectively. Similarly, using RD DI, 13 and 47% of the cases were accurately estimated with the one- and two-box models (Figure 2c,d and Table 2). Thus, with the selected parameter settings, for the inhalable mass fraction, more accurate modelling results were obtained using the RD D, whereas for the respirable mass fraction slightly better results were obtained using the CD DI.

Modelled concentrations were seen to have a direct and consistent correlation with the DI. This can be clearly observed for the one- and two-box modelled respirable mass concentrations, which were generally higher when using RD DI than the CD DI, except for Kaolin 1, Feldspar 2 and Kaolin 2, all of these materials showing a lower RD DI (Figure 2c,d and Table 1). The large difference obtained for modelled inhalable concentrations when using CD and RD DI as input (large overestimations using CD DI opposed to more accurate estimations using RD) is remarkable. This behaviour was not observed for respirable mass concentration. The ratio of respirable CD and RD DI for all materials is on average 1.24 (0.46–2.44). Conversely, for inhalable mass fraction the average ratio is 25.10 (17.09–53.50). Thus, to obtain similar modelled inhalable mass concentrations using the CD, the H factor should be 25 times smaller than 1 (0.04 for filling lines L and M) and 0.5 (0.02 for filling line H). When using these corrected H factors of 0.04 and 0.02, modelled concentrations with one- and two-box models ranged between 3.68–50.66 and 1.23–16.41 µg m^−3^, respectively with 46.7% and 86.7% of measured concentrations estimated with ranges between 0.5 and 5 with the one- and two-box models (data not shown). These observations show the need to continue studying how different dustiness test methods can be applied for modelling of powder handling scenarios and the importance of correct parametrization of the H factor, which is key for obtaining accurate modelling results, not only for the different dustiness methods but also fractions.

The particular difference observed for inhalable dust may be explained by the design of the CD dustiness test. Recently, Shandilya et al. [38] concluded that the two most influential properties on the DI from the CD were the average inter-particle distance (bulk density) and the drag force from the upward flow, which is characteristic of the process. The presence of this force during the CD dustiness test, which is not present in the RD and neither in a normal filling process, may be one of the possible explanations for the high overestimations of exposure obtained in this work when using the CD as input. The effect of this drag force most likely enable extended duration of the suspension of coarser and low effective density inhalable particles. This hypothesis and the effect of the drag force should be further studied and taken into account when using the CD DI as a base for emission source characterisation. Another factor adding to the general overestimation of inhalable mass fraction is the fact that deposition was not considered in the applied models. Deposition effects are known to be quite relevant especially for coarse particles [39,40]. However, first order estimates following Lai and Nazaroff [41] methodology, suggest that 1–2% of the particles in the range of 11.5 nm up to 35 µm, would have settled after 60 min in these environments, being ventilation the dominating process for particle removal. Therefore, the deposition effect alone do not explain the remarkable difference between the inhalable DI values for the CD as compared to the RD method. This shows the importance of determining size-resolved concentrations during dustiness testing, which can be key for accurate modelling.

### 3.2. Model Performance Comparison between One- and Two-Box Models

Significant differences in predicted dust concentrations depending on the use of the one- or the two-box model. When using the two-box model the intra- and inter- material variability was reduced (Figure 2), and both, accuracy and precision of the results were improved for all materials and mass fractions studied (Table 2 and Figure 2).

As expected from the DI results above, both, the one- and two-box models highly overestimated the inhalable particle mass concentrations when using the CD DI as input parameter under the specific settings selected (ratio > 40 one-box model, >17 two-box model) (Figure 2a), with a MAE of 314.1 and 108.9 (Supplementary Table S3). On the other hand, when using RD DI, monitored concentrations were modelled with ratios < 5 in 53–87% with a MAE of 8.7 and 2.0 using the one- and two-box models, respectively (Figure 2b; Supplementary Table S3). The respirable mass fraction was accurately estimated in 27 and 67% with a MAE of 1.0 and 0.27 of the cases using the CD DI with the one- and two-box models, respectively (Figure 2c; Supplementary Table S4). Similarly, using the RD DI monitored concentration were accurately estimated in 13 and 47% with a MAE of 0.85 and 0.24 of the cases using the one- and two box models (Figure 2d; Supplementary Table S4). The models underestimated the respirable mass fraction (ratio < 0.5) in only one occasion. However, ratios between 0.99–0.5 were obtained for 1 out of 15 cases when using the one-box model with RD DI and, in 3 and 4 out of 15 cases when using the two-box model with CD and RD DI, respectively. Even though this would be acceptable considering the benchmark used in this work, large underestimations of exposure mass-concentrations are never desirable for risk management purposes and therefore care should be taken when applying these models.

The use of the two-box model clearly improved model performance by means of accuracy and precision in a consistent way for all studied cases. This is in concordance with previous results from several authors where the one-box model was observed to underestimate NF concentrations while overestimating FF concentrations [8,10]. In Jensen et al. [10], the box modelling performance was improved when adding n-boxes to the model (2-box and 3-box). However, the one-box model, even with its simplified assumptions provided quite accurate results for the cases under study. These results are also in concordance with the ones presented in [13] where good modelling performance of the one- and two-box models were obtained for filling of a fertilizer, with a slightly better performance of the two-box model. It is important to note that room size and air mixing of the case under study play an important role on modelling performance, and for well-mixed small to medium size rooms the one-box model has shown to perform well.

In general terms, and considering the apparent model performance for the respirable mass fraction (53 and 87% of the cases were modelled within 0.5–5 ratio range with the one- and two-box models, respectively), it may be concluded that, with DI based emission characterization and adequate parametrization, the one- and two-box models may provide useful guidance regarding the order of magnitude of expected mass-based particle exposure levels. Conversely, DI based inhalable emission source modelling should be further studied.

In an attempt to make the one- and two-box models assessment comparable to the assessments conducted for several exposure assessment tools such as Stoffenmanager and ART, R^2^ and Spearman correlation coefficient were calculated (Supplementary Figures S7 and S8). The calculated R^2^ between measured and modelled concentrations with the one- and two-box models was <0.18 for the respirable concentrations, whereas Spearman correlation ranged from −0.20 to 0.24. Conversely, for the inhalable mass fraction, R^2^ of 0.50 and 0.44 were obtained for the one- and two-box models, respectively when using the CD DI, and R^2^ < 0.28 was observed when RD DI was used. Spearman correlations ranged from −0.11 to 0.25. These results are far from the criteria proposed in Fransman et al. [37] of a Spearman correlation > 0.6. However, it is important to keep in mind that (1) a low number of data points was used (<20), (2) the data was clustered, and (3) the concentration range is limited and not widely spread (See Supplementary Figures S7 and S8). Thus, in this specific case, care should be taken when interpreting these results as, if only looking at the R^2^ and Spearman correlations one could have the impression that modelled concentrations for the inhalable fraction are in better agreement with measured concentrations than for the respirable fraction, which is not true. This shows that when assessing exposure models performance, it is highly relevant to use data sets, which are relatively large and with widely spread data points across the possible range.

The measured respirable mass fraction exceeded modelled respirable concentrations in more than 10% of the cases when using RD DI with the one- and two-box model (40 and 13.3%) and the CD DI with the two-box model (26.7%). Inhalable measured mass fractions exceeded modelled inhalable mass fraction only when using RD DI and the two-box model (13.3%). However, in any case measured concentrations were more than double than modelled concentrations.

### 3.3. Effect of Background Concentrations on Modelled Respirable Mass

**Figure 3:**
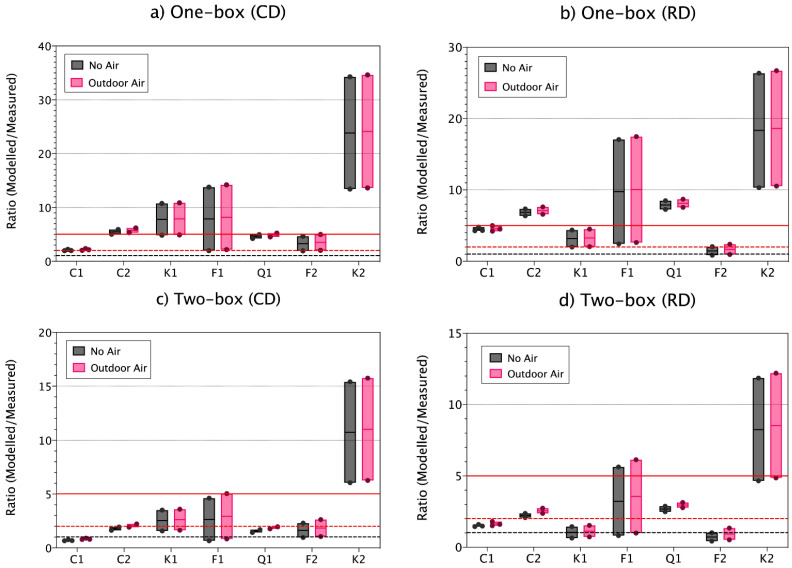
Vertical boxplot for ratio modelled/measured respirable concentrations for all materials without including incoming concentrations and including outdoor concentrations when using (**a**) CD as input for the one-box model, (**b**) RD as input for the one-box model, (**c**) CD as input for the two-box model and (**d**) RD as input for the two-box model. Ratios 1, 2 and 5 are marked as reference (red solid line, red dashed line and black dashed line, respectively). Solid coloured line within the box indicates the median value, and the limits of the box indicate min and max values. Coloured dots represent the individual replicate values. Modelling parameters: LEV and β fixed at 70% and 5 m^3^ min^−1^, respectively.

The effect of background air on modelled concentration was studied given that results from previous work indicated that this can improve model performance and accuracy [14]. In the current study, inclusion of background air mass-concentrations had only low impact on modelled inhalable and respirable concentrations (Figure 3), even though the workplace air was affected by other background activities including diesel-powered forklifts. Thus, including outdoor concentration seems to be in this case less crucial and in contrast to findings in Ribalta et al. [13,36].

### 3.4. Effect of LEV Reduction on Modelled Respirable Concentrations

The use of LEV and other local controls to reduce worker exposure in occupational environments is a common practice in industrial settings. In real-world scenarios it is sometimes hard to obtain precise reduction efficiency values for local controls.

**Figure 4:**
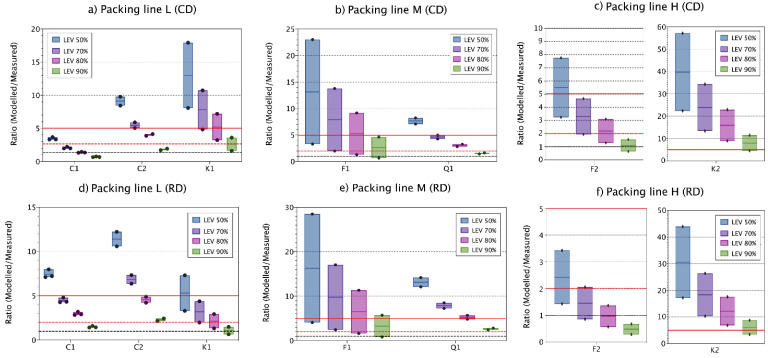
Vertical boxplot for ratio one-box modelled/measured respirable concentrations for all materials when using CD DI (**a**–**c**) and RD DI (**d**–**f**) as input parameter for source characterisation. Ratios 1, 2 and 5 are marked as reference (red solid line, red dashed line and black dashed lines, respectively). Solid coloured line within the box indicates the median value, the limits of the box indicate min and max values. Coloured dots represent the individual replicate values. Modelling parameters: β_i_ fixed at 5 m^3^ min^−1^.

Using different LEV reduction percentages had significant effects on modelled respirable concentrations, with variations on the concentrations up to 80% depending on the LEV value used (50–90% reduction) for, the one- and two-box models and independently of the DI used (CD or RD) (Figure 4).

Respirable measured concentrations with one- and two-box models were quite accurately predicted for filling line L and M considering all materials, especially when using LEV reductions of 70, 80 and 90% percent for both, the one- and two-box models and independently of the dustiness methods, but with slightly better results when using the two-box model and the RD DI. Conversely, for filling line H, modelled concentrations were highly overestimated and high variability between repetitions and materials was observed (Figure 4). When using 50% LEV reduction, one- and two-box model concentrations overestimated monitored concentrations (ratio > 2) by 67 up to 100%, whereas using 70-, 80- and 90%-LEV reductions, accurately estimated 13–67%, 27–67% and 47–73% of the cases, respectively. Thus, as expected, LEV reduction values used for modelling can have a major impact on modelled concentrations. However, small variations between 10–20% LEV differences should be acceptable as even though exposure concentrations are different, they lead to similar conclusions.

**Figure 5:**
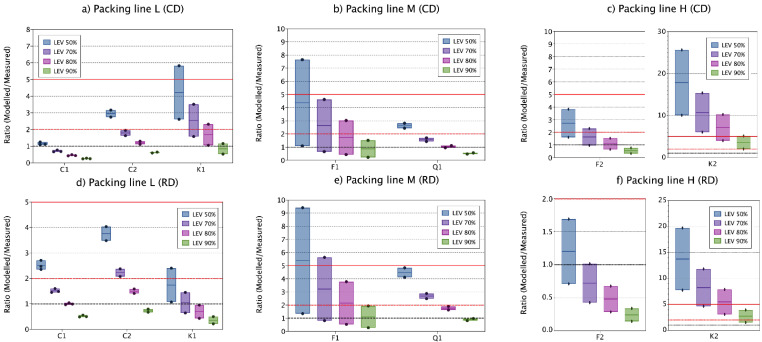
Vertical boxplot for ratio two-box modelled/measured respirable concentrations for all materials when using CD DI (**a**–**c**) and RD DI (**d**–**f**) as input parameter for source characterisation. Ratios 1, 2 and 5 are marked as reference (red solid line, red dashed line and black dashed lines, respectively). Solid coloured line within the box indicates the median value, and the limits of the box indicate min and max values. Coloured dots represent the individual replicate values. Modelling parameters: β_i_ fixed at 5 m^3^ min^−1^.

Modelled results for filling line L were the most accurate and precise for both, one- and two-box models, and independently of the DI used (Figure 4 and Figure 5, and Table 2). For filling lines L and M (with a total of 5 materials with 2–3 repetitions for material), modelled respirable concentrations were in general quite accurate and precise, indicating an adequate parametrization of the modelled scenarios (Figure 4a,b,d,e and Figure 5a,b,d,e). However, Kaolin 1 (line L) and Feldspar 1 (line M) showed low precision, with high intra-material variability. However, this ratio variability is not related to the modelled concentrations (Table 2) but to the monitored exposure concentrations, as during these cases, exposure concentrations were influenced by sources other than filling [30].

On the other hand, and as stated above, for filling line H, predicted concentrations showed lower precision and high overestimations of measured concentrations. However, when looking at the ratios individually for each material (Figure 4c,f and Figure 5c,f), Feldspar 2 ratios coincided with ratios obtained for filling line L and M, with accurate estimations, whereas Kaolin 2 modelled concentrations highly overestimated measured concentrations (Figure 4c,f and Figure 5c,f). During filling of Feldspar 2 several incidents during the process of filling occurred (e.g., bags broken during filling). These incidents were recorded and seen to have an important impact on measured concentrations. On the other hand, during filling of Kaolin 2, no effects on exposure concentrations were monitored [30]. When these punctual events were removed from the measured concentrations, ratio behaviour for Feldspar 2 was closer to Kaolin 2 (Table 2), showing high overestimation of inhalable mass fraction. However, for respirable mass fractions, Feldspar 2 mass concentrations were predicted with ratios < 5 in 87.5% of the cases versus 12.5% for Kaolin 2. For medium level containment, a median and maximum efficacy of 95 and 100% is reported in the ECEL library (see Supplementary Section S3, Figures S3 and S4). For precautionary reasons, 90% was selected here. However, the obtained results suggests that the LC_bag_ real value is probably higher than the selected (90%) and thus the better fit of the modelled concentrations for Feldspar 2 for which unexpected events occurred during filling (e.g., broken bags during filling). The one- and two-box models are developed for constant and cyclic emissions, which does not exactly correspond with the type of scenario in filling of Feldspar 2 in line H. Thus, this shows the relevance to consider also likely accidents in the modelling approach.

### 3.5. Effect of Inter-Zonal Flows (β) on Modelled Respirable Concentrations

Inter-zonal NF-FF air flow, also so called β, is one of the most complex exposure model parameters to determine as it is driven by complex air current interactions, the movement and heat from the workers equipment and local controls, among other factors [6,26,42]. For this reason, the analysis of the effect of β on modelling performance output is paramount.

**Figure 6:**
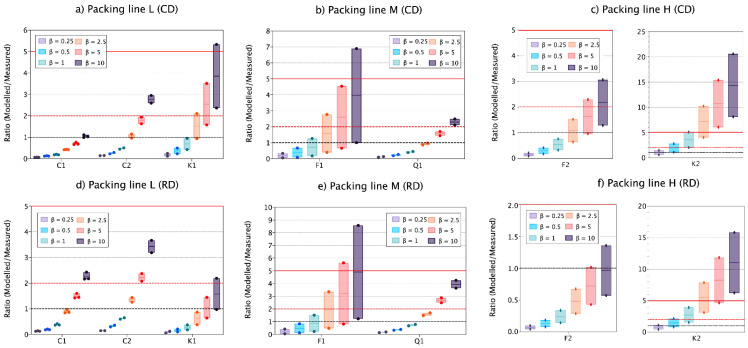
Vertical boxplot for ratio modelled/measured respirable concentrations for all materials when using CD DI (**a**–**c**) and RD DI (**d**–**f**) using RD DI as input parameter for source characterisation. Ratios 1, 2 and 5 are marked as reference (red solid line, red dashed line and black dashed lines, respectively). Solid coloured line within the box indicates the median value. Modelling parameters: LEV and fixed at 70%.

Clear effects on modelled respirable concentrations due to β changes were observed for all filling lines (Figure 6). A common and gradual trend was observed with the increase of β for all materials and filling lines. In filling line L and M, β values of 0.25–1 m^3^ min^−1^ underestimated monitored concentrations in 82 and 88% of the cases when using RD and CD DI. Conversely, β values of 2.5–10 m^3^ min^−1^ accurately estimated measured concentrations on 48–55% and slightly overestimated 27–39% of the cases (Figure 6). For filling line H, as previously described for LEV variations, high intra-filling line variability was observed, with Feldspar 2 ratio of modelled/measured concentrations showing a similar behaviour to materials in lines L and M, and Kaolin 2 ratios highly overestimating independently of the β values used (Figure 6).

For Clay 1, two-box modelled CD concentrations generally underestimated monitored exposures when β values 0.25–2.5 m^3^ min^−1^ were used, and accurately estimated exposures when using β values of 5 and 10 m^3^ min^−1^. Conversely, monitored concentrations for Clay 2 and Kaolin 1 were accurately and precisely estimated when using β 1–5 m^3^ min^−1^ (Figure 6a), with ratios modelled/measured between 0.5–1.9 and 0.9–1.6 for Clay 2 and Kaolin 1, respectively. In filling line M, and for both materials, most accurate values were obtained when using β values from 1–5 m^3^ min^−1^ (Figure 6b). Conversely, in filling line H, Feldspar 2 monitored concentrations were predicted accurately inside the 0.5–2 benchmark for β values 2.5–10 m^3^ min^−1^, whereas for Kaolin 2, ratios modelled/measured were always >5 for those β values (Figure 6c).

On the other hand, two-box modelled RD concentrations for all materials packed in line L were accurately estimated when using β values between 2.5 and 10 m^3^ min^−1^ and a lower intra-filling line response to β value changes was observed compared to CD modelling results (Figure 6d). This effect was also observed, although less strong, for filling line M (Figure 6e). Finally, for filling line H, again high variability was observed between materials, with Feldspar 2 showing more accurate results with β values of 2.5–10 m^3^ min^−1^ whereas for Kaolin 2 with β values 0.5–2.5 m^3^ min^−1^ (Figure 6f).

As with LEV, different β values significantly changed modelling results and outcomes. However, 2- to 5-fold increases between β values (e.g., 2.5–5 m^3^ min^−1^) should not lead to large differences on modelling outcomes. Strong effects on modelling outcomes when using different values of β have been previously reported for different environments such as industrial scenarios, chamber experiments or medical sites [10,11,13,43]. Therefore, efforts on measuring inter-zonal airflows for modelling are paramount, even though their exact characterisation is complex.

## 4. Conclusions

The performance, applicability and robustness of the one- and two-box models under real-world industrial scenarios (filling of powdered materials) was evaluated. A previously published case study where seven materials packed in three filling lines with different levels of energy and mitigation strategies applied was used [30]. Two different DI methods were used (CD and RD). Exposure concentrations were modelled in terms of inhalable and respirable mass fractions, and effects of different LEV and β values were analysed. In addition, the effect of background concentrations on modelled mass-concentrations was studied.

Model performance was strongly impacted by the choice of the DI method and scaling factor applied. Inhalable mass was highly overestimated with the one- and two-box models when using CD method (ratios > 15). Conversely, using RD DI methods, model performance significantly improved. This result was unexpected as the CD method is considered to resemble processes such as feeding and pouring of powder more closely than the RD method. This observation was impacted by the used H value, which was the same for both methods. However, for the respirable mass fraction both dustiness tests provided similar results. The differences between DI determined by the CD versus the RD may in part be caused by the presence of an upward moving flow in the dustiness test column which introduces a drag force to the aerosol during the determination of the CD DI. This may force some slowed settling of dust particles released. This force is likely to have a stronger effect on inhalable fraction than respirable, thus the difference on modelling performance. This shows that the effective dispersion during the dustiness test is higher than during the process, and shows the need to further understand how the different dustiness methods relate to specific processes and to standardize H values for different dustiness methods, process and mass fractions, which is key for modelling and exposure assessment.

Including outdoor concentrations did not improve the one- and the two-box model performance in the scenarios analysed, as exposure concentrations were mainly driven by the filling process. Therefore, the decision of including or not background concentrations should be taken according to each specific case.

Strong impacts on model outcomes were observed depending on the LEV and β values used. Using 70- or 90%-LEV values can lead from under- to overestimation of particle mass concentration. However, differences of 10–20% of LEV reduction provided similar modelling outcomes. Similarly, using different β values provided from under- to overestimations of monitored exposure concentrations although a certain degree of imprecision (two- to five-fold changes) would not lead to incorrect decision making. These limits need to be understood.

Finally, in the tested cases (which had room volumes ranging from 420 to 2100 m^3^), modelled concentrations were more accurate and precise when using the two-box model than the one-box model, with one-box generally overestimating worker area monitored concentrations. The one-box model estimated 53% of the cases within the 0.5–5 ratio whereas the percentage increased up to 87% for the two-box model. The one-box model may be useful for simple scenarios with good air mixing, but for complex scenarios including enclosures and LEV systems, the use of a two-box model may provide more accurate and precise results. In summary, both, the one- and two-box models, when using DI as input parameter for the source emission characterization, were seen to accurately and precisely estimate respirable mass concentrations for different scenarios in a quite robust way if adequate parametrization for the given scenarios were applied. However, further understanding on how to scale dustiness to process by means of the H factor is needed. Additionally, studies are needed to identify most appropriate values for the determinant model parameters to improve the general model performance. Finally, it was shown how the use of the one- and two-box models for unexpected events should be conducted with care, by clearly identifying and determining specific emission rates for the different conditions or the results could lead to impaired decision making.

## Figures and Tables

**Table 1:** Variables used in Equation 1. Dustiness index (CD and RD) for material, calculated dM/dt, H factor and LC due to the effect of enclosure of the bag.

Filling Line	Material	Equation (1) Variables (Unit)						
		Continuous Drop DI (mg kg^−1^)		Rotating Drum DI (mg kg^−1^)		dM/dt (kg min^−1^)	H (−)	LC_bag_ (−)
		W_I_ ± SD	W_R_ ± SD	W_I_ ± SD	W_R_ ± SD			
L	Clay 1	1733 *	6 *	96 *	13 *	800	1	0.3 (70% reduction)
	Clay 2	5170 **	16 *	192 *	20 *	600	1	
	Kaolin 1	18,886 ***	44*	353 *	18 *	850	1	
M	Feldspar 1	10,246 **	59 *	455 *	73 **	530	1	0.2 (80% reduction)
	Quartz 1	8891 **	43 *	480 *	75 **	550	1	
H	Feldspar 2	9651 **	77 **	505 *	34 *	100–250	0.5	0.1 (90% reduction)
	Kaolin 2	12,325 **	104 **	721 **	80 **		0.5	

* Low and very low dustiness index; ** medium dustiness index; *** high dustiness index according to EN 15051 classification for CD and RD dustiness methods, and inhalable and respirable fractions. SD: arithmetic standard deviation.

**Table 2:** Measured stationary concentrations at the worker area, and one- and two-box modelling results for inhalable and respirable mass fractions (mg m^−3^), and for CD and RD dustiness index. Ratio modelled/measured shown in brackets. Modelling parameters: LEV and β fixed at 70% and 5 m^3^ min^−1^, respectively. Rn: number of replicates for each material. * Not considering unexpected events.

			Filling Line L							Filling Line M				Filling Line H			
Mass Fraction	Model	DI Method	Clay 1			Clay 2		Kaolin 1		Feldspar 1		Quartz 1		Feldspar 2		Kaolin 2	
			R1	R2	R3	R1	R2	R1	R2	R1	R2	R1	R2	R1	R2	R1	R2
Inhalable	One-box	CD	92.1 (50)	94.5 (56)	93.4 (68)	263.6 (132)	221.2 (143)	1117 (422)	1266.5 (269)	198.4 (58)	285.1 (201)	155.2 (91)	181.8 (158)	170.9 (40/174 *)	168.7 (107/129 *)	217.9 (263)	215.3 (761)
		RD	5.1 (2.8)	5.2 (3.1)	5.2 (3.8)	9.8 (4.9)	8.2 (5.3)	20.9 (7.9)	23.7 (5.0)	8.8 (2.6)	12.7 (9.0)	8.3 (4.8)	9.7 (8.4)	8.9 (2.1/9.1 *)	8.8 (5.6/6.8 *)	12.8 (15)	12.6 (45)
	Two-box	CD	30.9 (17)	31.5 (19)	31.3 (23)	86.1 (43)	72.3 (47)	362.9 (137)	410.1 (87)	65.9 (19)	94.6 (67)	52.7 (31)	61.8 (54)	84.8 (20/86.5 *)	83.2 (53/64.0 *)	98.3 (118)	96.8 (342)
		RD	1.7 (0.9)	1.8 (1.0)	1.7 (1.3)	3.2 (1.6)	2.7 (1.7)	6.8 (2.6)	7.7 (1.6)	2.9 (0.9)	4.2 (3.0)	2.8 (1.6)	3.3 (2.9)	4.4 (1.0/4.5 *)	4.4 (2.8/3.4 *)	5.8 (6.9)	5.7 (20)
	Measured	-	1.85	1.70	1.37	2.00	1.54	2.65	4.71	3.42	1.41	1.71	1.15	4.26/0.98 *	1.57/1.30 *	0.83	0.28
Respirable	One-box	CD	0.32 (2.2)	0.33 (2.0)	0.32 (2.0)	0.82 (5.9)	0.68 (5.0)	2.6 (11)	3.0 (4.8)	1.1 (2.0)	1.6 (14)	0.76 (5.0)	0.89 (4.3)	1.4 (1.9/8.0 *)	1.4 (4.7/4.7 *)	1.8 (13)	1.8 (34)
		RD	0.69 (4.8)	0.71 (4.3)	0.70 (4.3)	1.0 (7.3)	0.86 (6.4)	1.1 (4.4)	1.2 (2.0)	1.4 (2.4)	2.0 (17)	1.3 (8.5)	1.5 (7.3)	0.60 (0.9/3.5 *)	0.59 (2.1/2.0 *)	1.4 (10)	1.4 (26)
	Two-box	CD	0.11 (0.8)	0.11 (0.7)	0.11 (0.7)	0.27 (1.9)	0.22 (1.6)	0.85 (3.5)	0.96 (1.6)	0.38 (0.7)	0.55 (4.6)	0.26 (1.7)	0.30 (1.4)	0.68 (1.0/4.0 *)	0.66 (2.3/2.3 *)	0.83 (6.1)	0.82 (15)
		RD	0.23 (1.6)	0.24 (1.4)	0.24 (1.5)	0.33 (2.4)	0.28 (2.1)	0.35 (1.4)	0.39 (0.6)	0.47 (0.8)	0.67 (5.6)	0.44 (2.9)	0.52 (2.5)	0.30 (0.4/1.8 *)	0.29 (1.0/1.0 *)	0.64 (4.7)	0.63 (12)
	Measured	-	0.14	0.17	0.16	0.14	0.14	0.24	0.61	0.58	0.12	0.15	0.21	0.70/0.17 *	0.29/0.26 *	0.14	0.05

## Data Availability

Data are available from the corresponding author upon request.

## Supplementary Materials

The following are available online at https://www.mdpi.com/article/10.3390/toxics9090201/s1, Table S1: materials for filling line and code, number of batch repetitions, powdered material particle size and moisture content, and environment characteristics, total air flow (Q m^3^ min^−1^) and corresponding air changes per hour (ACH h^−1^), Table S2: ranking categories for continuous drop (CD) and rotating drum (RD) dustiness methods according to the EN 15051, Table S3: Difference between modelled and measured inhalable concentration (Diff.), absolute difference (Abs. diff.) and mean absolute error (MAE) for each model run. C1: Clay 1; C2: Clay 2; K1: Kaolin 1; F1: Feldspar 1; Q1: Quarts 1; F2: Feldspar 2; K2: Kaolin 2, Table S4: Difference between modelled and measured respirable concentration (Diff.), absolute difference (Abs. diff.) and mean absolute error (MAE) for each model run. C1: Clay 1; C2: Clay 2; K1: Kaolin 1; F1: Feldspar 1; Q1: Quarts 1; F2: Feldspar 2; K2: Kaolin 2, Figure S1: Search selection of risk management measures (RMM) percentage reductions for tasks (bagging/dumping/filling, packing/bottling, transfer powders, transfer during packing and pouring of powders) in ECEL. Source: screenshot from https://diamonds.tno.nl/#ecel, accessed on 4 June 2021, Figure S2: Overview of percentages of reduction due to different risk management measures (RMM) on process selection from Figure S1. Source: screenshot from https://diamonds.tno.nl/#ecel, accessed on 4 June 2021, Figure S3: Search selection for risk management measures (RMM) percentage reductions due to isolation/segregation, containment without ventilation, low-level containment, not specified segregation and medium level containment on general tasks in ECEL. Source: screenshot from https://diamonds.tno.nl/#ecel, accessed on 4 June 2021, Figure S4: Overview of percentages of reduction due to due to isolation/segregation, containment without ventilation, low-level containment, not specified segregation and medium level containment on general processes. RMM: risk management measures. Source: screenshot from https://diamonds.tno.nl/#ecel, accessed on 4 June 2021, Figure S5: Search selection of “fixed capturing hoods” risk management measure on bagging, dumping, filling, packing/bottling, transfer of powders, transfer during packing and pouring of powders in ECEL. Source: screenshot from https://diamonds.tno.nl/#ecel, accessed on 4 June 2021, Figure S6: Overview of percentages of reduction due to due to “fixed capturing hoods” on process selection from Figure S5. RMM: risk management measures. Source: screenshot from https://diamonds.tno.nl/#ecel, accessed on 4 June 2021, Figure S7: Linear regression, R2 and Spearman’s correlation coefficient (c.c.) for respirable modelled concentration and measured concentrations when using (a) one-box model and CD DI, (b) one-box model and RD DI, (c) two-box model and CD DI, and (d) two-box model and RD DI, Figure S8: Linear regression, R2 and Spearman’s correlation coefficient (c.c.) for inhalable modelled concentration and measured concentrations when using (a) one-box model and CD DI, (b) one-box model and RD DI, (c) two-box model and CD DI, and (d) two-box model and RD DI.
