# Supplementary material for: Evaluation of One- and Two-Box Models as Particle Exposure Prediction Tools at Industrial Scale
Source: Toxics. 2021 Aug 29;9(9):201. doi: 10.3390/toxics9090201 (PMC8471509; doi:10.3390/toxics9090201)
Supplement: Supplementary file 1 [file toxics-09-00201-s001.zip › toxics-1319624-supplementary - final1.pdf]

toxics MDPI

Supplementary Materials: Evaluation of One- and Two-Box Models as Particle Exposure Prediction Tools at Industrial Scale

Carla Ribalta, Ana López-Lilao, Ana Sofia Fonseca, Alexander Christian Østerskov Jensen, Keld Alstrup Jensen, Eliseo Monfort and Mar Viana

1. Materials and Work Environment Characteristics

Table S1. Materials for packing line and code, number of batch repetitions, powdered material particle size and moisture content, and environment characteristics, total air flow (Q m^3 min^{-1}) and corresponding air changes per hour (ACH h^{-1}).

Packing Line	Material	Code	Repetitions	Material Characteristic		Environment	
				d_{50} (μm)	Moisture (%)	ACH (h^{-1})	Q (m^3 min^{-1})
L	Clay 1	C1	3	13	11	21	746
	Clay 2	C2	2	10	13	12	415
	Kaolin 1	K1	2	13	11	12	415
M	Feldspar 1	F1	2	31–39	0.2	18	617
	Quartz 1	Q1	2	30–38	0.1	17	602
H	Feldspar 2	F2	2	22	0.3	29	206
	Kaolin 2	K2	2	8	0.7	32	227

2. Dustiness Results

Dustiness index results are expressed in mg kg^{-1} as well as in ranking categories according to the EN 15051:2013 classification. The ranking categories are described in Table S2.

Table S2. Ranking categories for continuous drop (CD) and rotating drum (RD) dustiness methods according to the EN 15051:2013.

mg kg^{-1}	Continuous Drop		Rotating Drum	
	Respirable	Inhalable	Respirable	Inhalable
Very low	<20	<1000	<10	<300
Low	20–70	1000–4000	10–60	300–650
Medium	70–300	400–15,000	60–210	650–3000
High	>300	>15,000	>210	>3000

Considering inhalable fraction from CD dustiness results, all materials were classified as medium DI, except Clay 1, with a low DI (1733 mg kg^{-1}) and Kaolin 1, with high DI (18,886 mg kg^{-1}), both materials being packed in Packing Line L. On the other hand, considering respirable fraction, all materials packed in Lines L and M were classified as materials with low dustiness index, and those packed in packing Line H, were classified as high dustiness index materials (Table 1). The material with higher respirable dustiness was Kaolin2, Packing Line H (104 mg kg^{-1}) and the lower Clay 1, Packing Line L (6 mg kg^{-1}).

According to RD dustiness results, all materials, except Kaolin 2, which was classified as medium DI, were classified as low DI when considering inhalable fraction. When considering respirable DI, all materials were classified as low, except Feldspar 1 and Quartz 1 (Packing Line M) and Feldspar 2 (Packing Line H).

3. Exposure Control Efficacy Library (ECEL v3.0) Search Results

This section provides the results from ECEL search (https://diamonds.tno.nl/#ecel, accessed on 14 June 2021) of reduction percentages due to risk management measures in place for different activities/tasks.

3.1. ECEL Reduction Efficacy of "Containment without Ventilation" on Bagging, Dumping, Filling, Packing/Bottling, Transfer of Powders, Transfer During Packing and Pouring of Powders

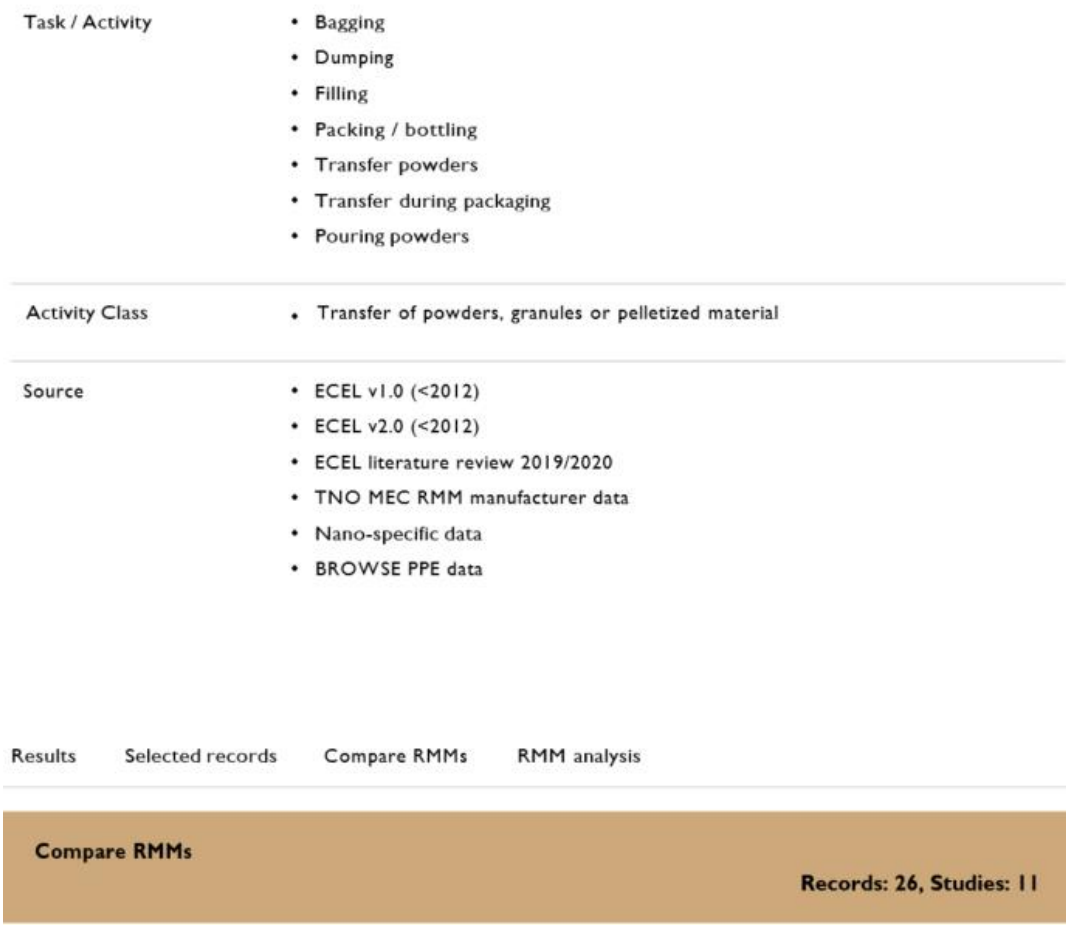

Figure S1. Search selection of risk management measures (RMM) reduction percentage for tasks (bagging/dumping/filling, packing/bottling, transfer powders, transfer during packing and pouring of powders) in ECEL. Source: screenshot from https://diamonds.tno.nl/#ecel, accessed on 14 June 2021.

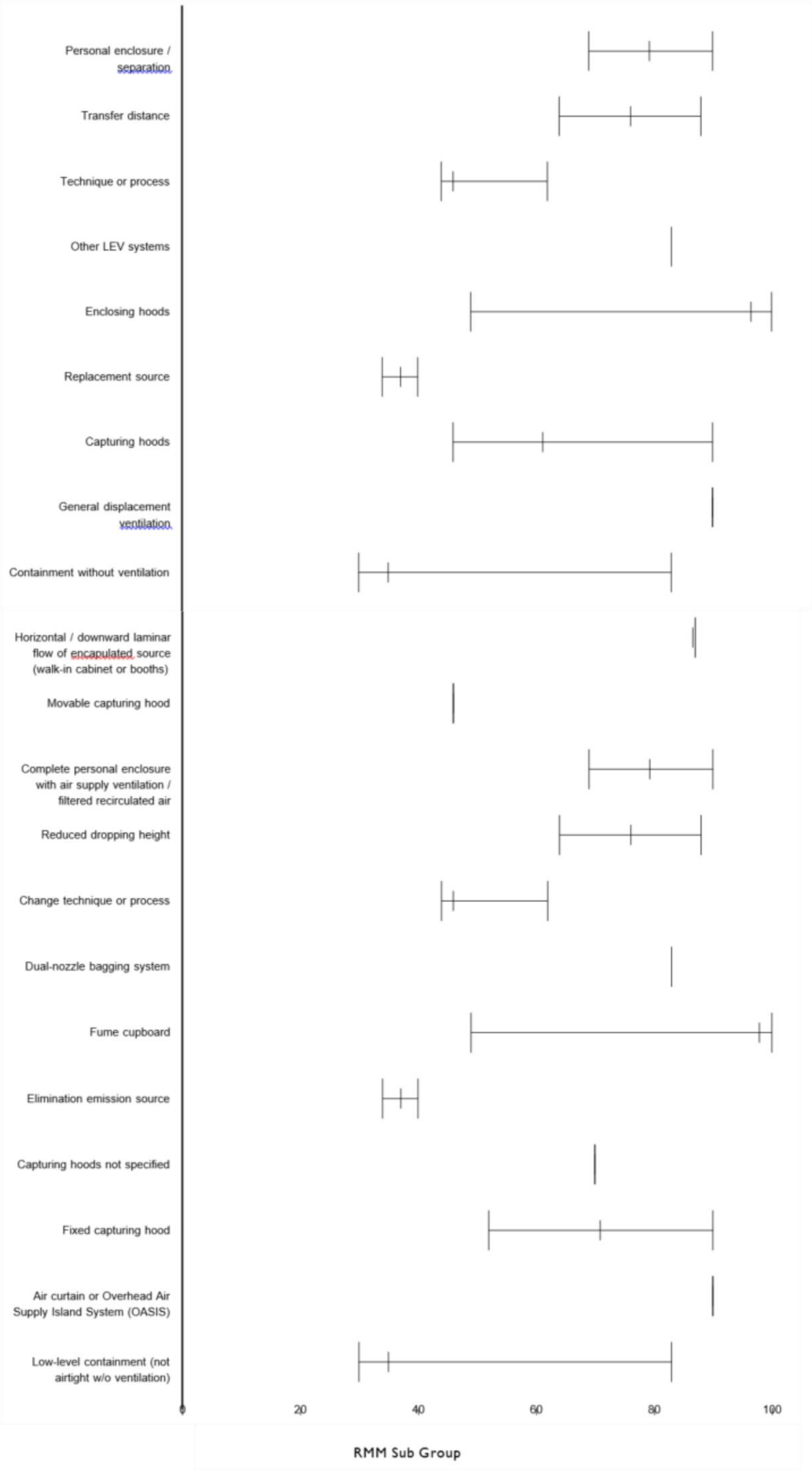
Personal enclosure / separation
Transfer distance
Technique or process
Other LEV systems
Enclosing hoods
Replacement source
Capturing hoods
General displacement ventilation
Containment without ventilation
Horizontal / downward laminar flow of encapulated source (walk-in cabinet or booths)
Movable capturing hood
Complete personal enclosure with air supply ventilation / filtered recirculated air
Reduced dropping height
Change technique or process
Dual-nozzle bagging system
Fume cupboard
Elimination emission source
Capturing hoods not specified
Fixed capturing hood
Air curtain or Overhead Air Supply Island System (OASIS)
Low-level containment (not airtight w/o ventilation)
0
20
40
60
80
100
RMM Sub Group

Figure S2. Overview of reduction percentages due to different risk management measures (RMM) on process selection from Figure S1. Source: screenshot from https://diamonds.tno.nl/#ecel, accessed on 14 June 2021.

3.2. ECEL Reduction Efficacy of "Low and Medium Level Containment" for General Processes

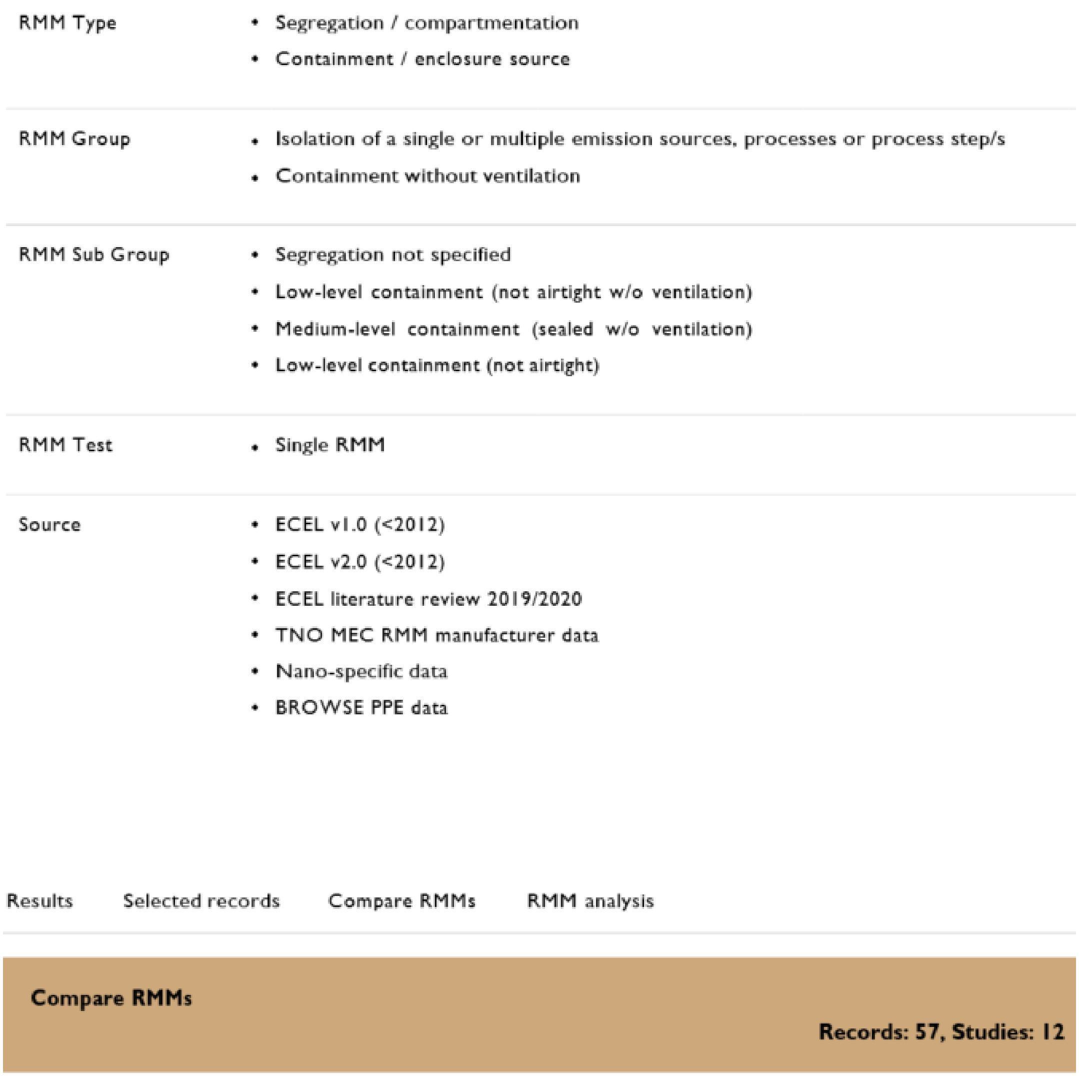

Figure S3. Search selection of risk management measures (RMM) reduction percentages due to isolation/segregation, containment without ventilation, low-level containment, not specified segregation and medium level containment on general tasks in ECEL. Source: screenshot from https://diamonds.tno.nl/#ecel, accessed on 14 June 2021.

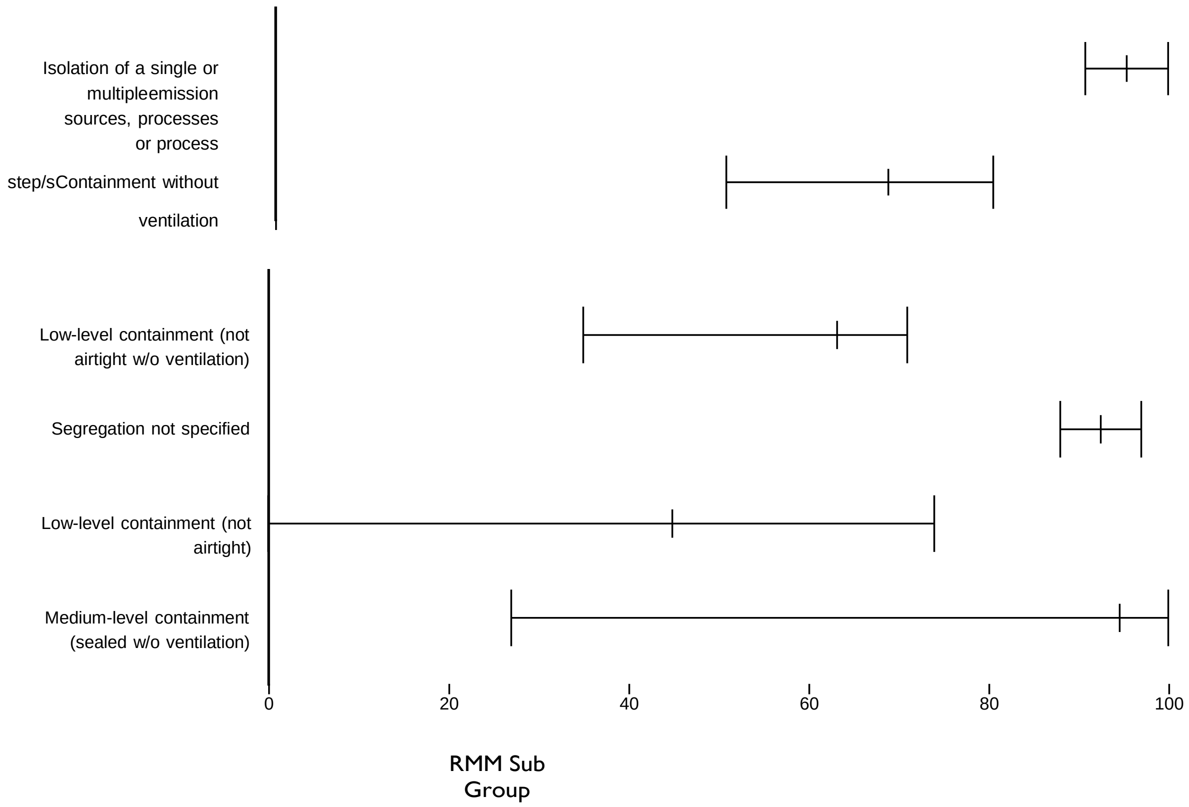

Figure S4. Overview of reduction percentages due to due to isolation/segregation, containment without ventilation, low-level containment, not specified segregation and medium level containment on general processes. RMM: risk management measures. Source: screenshot from https://diamonds.tno.nl/#ecel, accessed on 14 June 2021.

3.3. ECEL Reduction Efficacy of "Fixed Capturing Hoods" on Bagging, Dumping, Filling, Packing/Bottling, Transfer of Powders, Transfer During Packing and Pouring of Powders

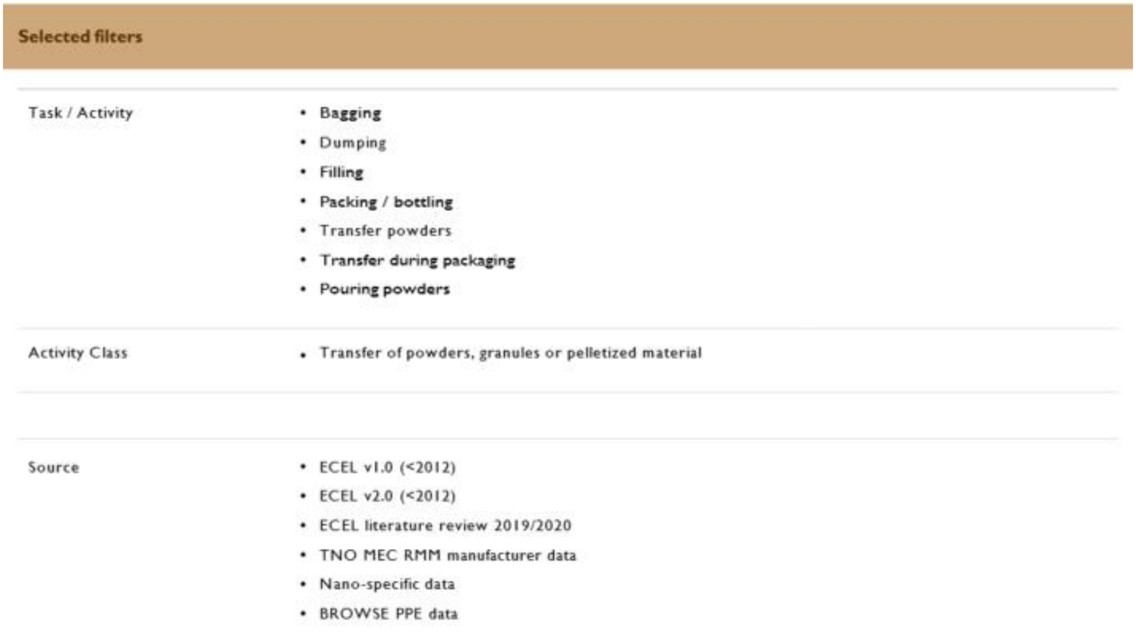

Figure S5. Search selection of "fixed capturing hoods" risk management measure on bagging, dumping, filling, packing/bottling, transfer of powders, transfer during packing and pouring of powders in ECEL. Source: screenshot from https://diamonds.tno.nl/#ecel, accessed on 14 June 2021.

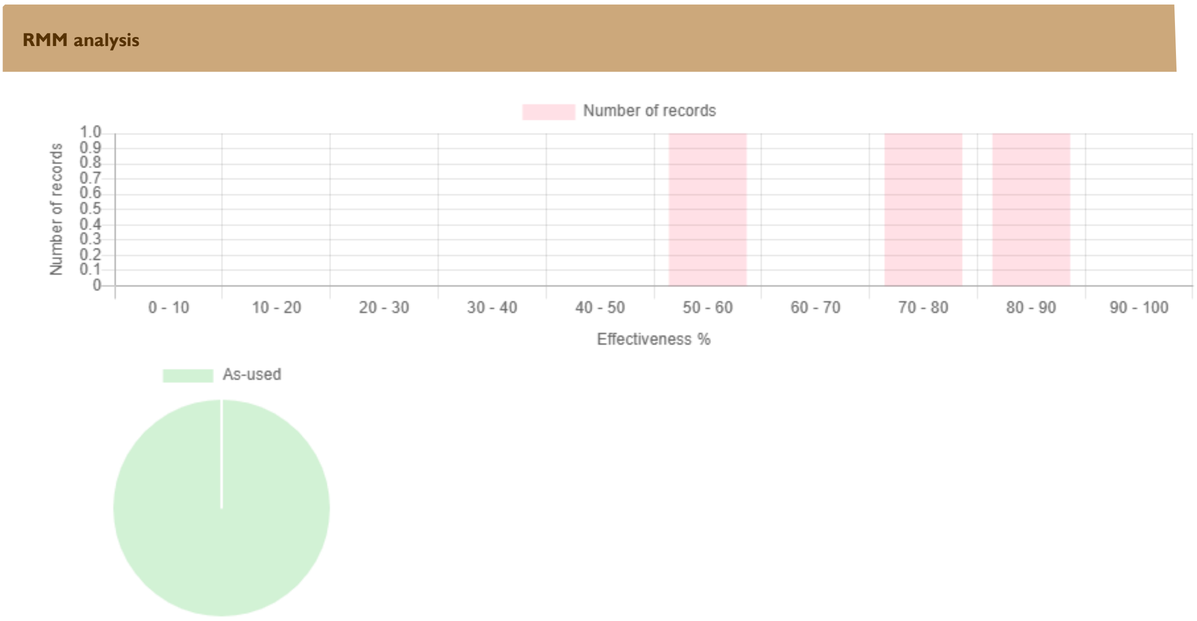

**Results**	**Value**
Number of available records	26
Number of selected records	3
Number of selected studies	2
Effectiveness Lower 95% HDI	52%
**Effectiveness Median**	**70%**
Effectiveness Upper 95% HDI	90%

Figure S6. Overview of reduction percentages due to due to "fixed capturing hoods" on process selection from Figure S5. RMM: risk management measures. Source: screenshot from https://diamonds.tno.nl/#ecel, accessed on 14 June 2021.

4. Monitored Mass Concentrations Results

Packing of materials was seen to increase inhalable and respirable exposure concentrations for most of the studied materials and were reported by (Ribalta et al., 2019). In summary, highest mean inhalable worker area/FF concentrations monitored were 4.7, 4.3 and 3.4 mg m^{-3} for Kaolin 1 (packing line L), Feldspar 2 (packing line H) and Feldspar 1 (packing line M), respectively. Similarly, highest respirable exposure concentrations were monitored for Feldspar 1 and 2, and Kaolin 1 with maximum mean concentrations between 0.70–0.57 mg m^{-3}. On the other hand, lowest inhalable and respirable concentration were monitored during packing of Kaolin 2 (packing line H), which is conversely one of the materials with higher DI, with concentrations of 0.28 and 0.053 mg m^{-3}, respectively.

5. Mean Absolute Error and Models Correlation

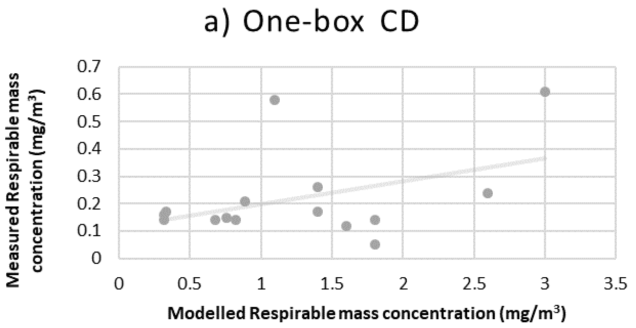

$y = 0.0849x + 0.1121$

$R^2 = 0.1821$

Spearman = 0.19

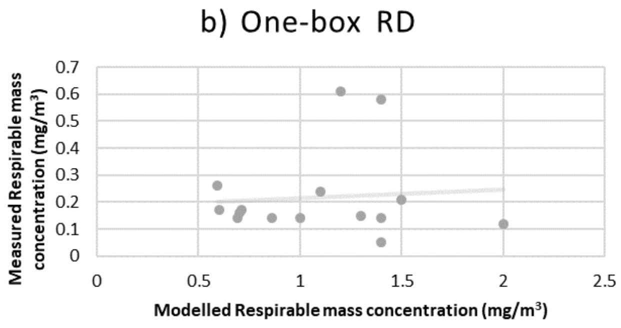

$y = 0.0307x + 0.185$

$R^2 = 0.0061$

Spearman = −0.21

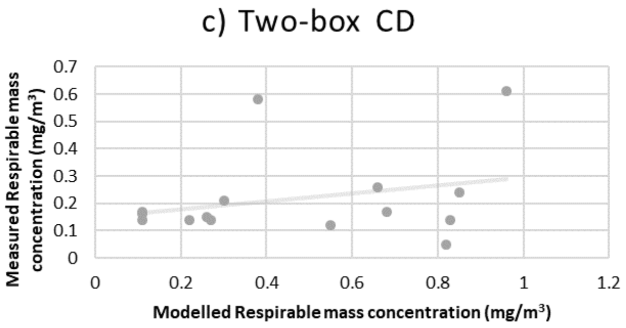

$y = 0.1451x + 0.1499$

$R^2 = 0.0754$

Spearman = 0.25

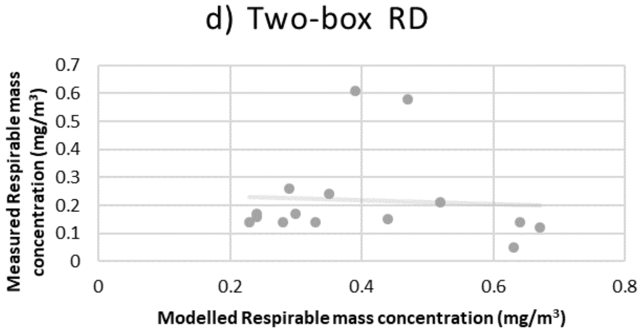

$y = -0.0669x + 0.2455$

$R^2 = 0.0041$

Spearman = −0.17

Figure S7. Linear regression, R^2 and Spearman's correlation coefficient (c.c.) for respirable modelled concentration and measured concentrations when using (**a**) one-box model and CD DI, (**b**) one-box model and RD DI, (**c**) two-box model and CD DI, and (**d**) two-box model and RD DI.

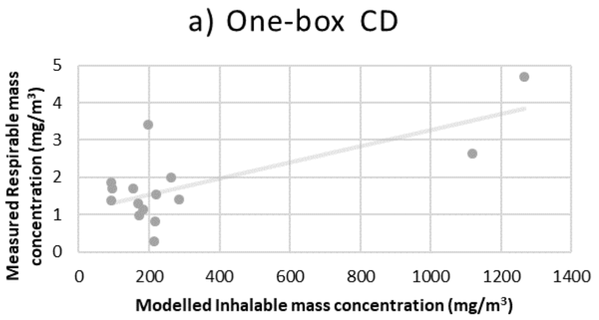

$y = 0.0022x + 1.1129$

$R^2 = 0.5015$

Spearman c.c. = 0.26

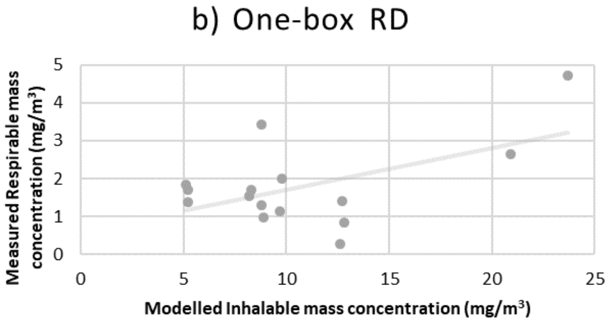

$y = 0.11x + 0.6153$

$R^2 = 0.2874$

Spearman c.c. = 0.007

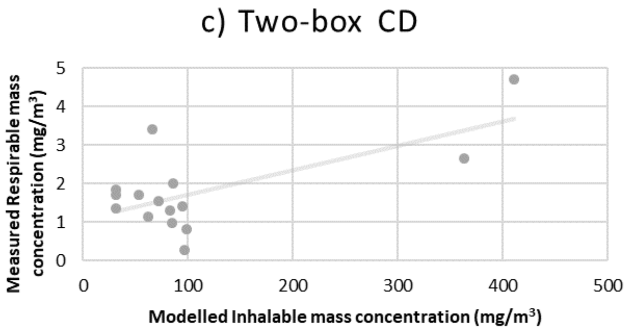

$y = 0.0063x + 1.09$

$R^2 = 0.4389$

Spearman c.c. = −0.004

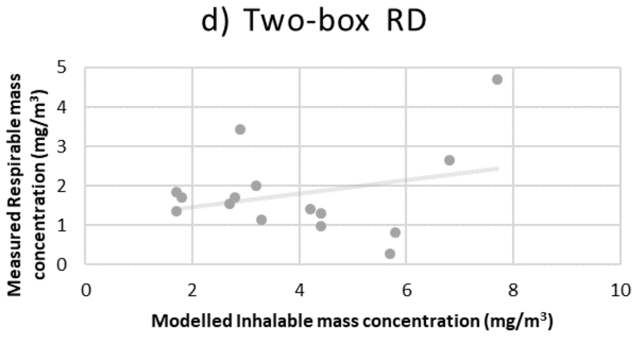

$y = 0.169x + 1.1274$

$R^2 = 0.0827$

Spearman c.c. = −0.12

Figure S8. Linear regression, R^2 and Spearman's correlation coefficient (c.c.) for inhalable modelled concentration and measured concentrations when using (**a**) one-box model and CD DI, (**b**) one-box model and RD DI, (**c**) two-box model and CD DI, and (**d**) two-box model and RD DI.

Table S3. Difference between modelled and measured inhalable concentration (Diff.), absolute difference (Abs. diff.) and mean absolute error (MAE) for each model run. C1: Clay 1; C2: Clay 2; K1: Kaolin 1; F1: Feldspar 1; Q1: Quarts 1; F2: Feldspar 2; K2: Kaolin 2.

Case	Measured (mg/m^3)	One-Box CD			One-Box RD			Two-Box CD			Two-Box RD		
		Model (mg/m^3)	Diff.	Abs. Diff.	Model (mg/m^3)	Diff.	Abs. Diff.	Model (mg/m^3)	Diff.	Abs. Diff.	Model (mg/m^3)	Diff.	Abs. Diff.
C1.R1	1.85	92.1	−90.3	90.3	5.1	−3.25	3.25	30.9	−29.1	29.1	1.7	0.15	0.15
C1.R2	1.70	94.5	−92.8	92.8	5.2	−3.50	3.50	31.5	−29.8	29.8	1.8	−0.10	0.10
C1.R3	1.37	93.4	−92.0	92.0	5.2	−3.83	3.83	31.3	−29.9	29.9	1.7	−0.33	0.33
C2.R1	2.00	263.6	−261.6	261.6	9.8	−7.80	7.80	86.1	−84.1	84.1	3.2	−1.2	1.2
C2.R2	1.54	221.2	−219.7	219.7	8.2	−6.66	6.66	72.3	−70.8	70.8	2.7	−1.2	1.2
K1.R1	2.65	1117.0	−1114	1114	20.9	−18.3	18.3	362.9	−360.3	360.3	6.8	−4.2	4.2
K1.R2	4.71	1266.5	−1262	1262	23.7	−19.0	19.0	410.1	−405.4	405.4	7.7	−3.0	3.0
F1.R1	3.42	198.4	−195.0	195.0	8.8	−5.38	5.38	65.9	−62.5	62.5	2.9	0.52	0.52
F1.R2	1.41	285.1	−283.7	283.7	12.7	−11.3	11.3	94.6	−93.2	93.2	4.2	−2.8	2.8
Q1.R1	1.71	155.2	−153.5	153.5	8.3	−6.59	6.59	52.7	−51.0	5.0	2.8	−1.1	1.1
Q1.R2	1.15	181.8	−180.7	180.7	9.7	−8.55	8.55	61.8	−60.7	60.7	3.3	−2.2	2.2
F2.R1	4.26	170.9	−166.6	166.6	8.9	−4.64	4.64	84.8	−80.5	80.5	4.4	−0.14	0.14
F2.R2	1.57	168.7	−167.1	167.1	8.8	−7.23	7.23	83.2	−81.6	81.6	4.4	−2.8	2.83

K2.R1	0.83	217.9	−217.1	217.1	12.8	−12.0	12.0	98.3	−97.5	97.5	5.8	−5.0	5.0
K2.R2	0.28	215.3	−215.0	215.0	12.6	−12.3	12.3	96.8	−96.5	96.5	5.7	−5.4	5.4
**MAE**			**314.1**			**8.7**			**108.9**			**2.0**	

Table S4. Difference between modelled and measured respirable concentration (Diff.), absolute difference (Abs. diff.) and mean absolute error (MAE) for each model run. C1: Clay 1; C2: Clay 2; K1: Kaolin 1; F1: Feldspar 1; Q1: Quarts 1; F2: Feldspar 2; K2: Kaolin 2.

**Case**	**Measured (mg/m^3)**	**One-Box CD**			**One-Box RD**			**Two-Box CD**			**Two-Box RD**		
		**Model (mg/m^3)**	**Diff.**	**Abs. Diff.**	**Model (mg/m^3)**	**Diff.**	**Abs. Diff.**	**Model (mg/m^3)**	**Diff.**	**Abs. Diff.**	**Model (mg/m^3)**	**Diff.**	**Abs. Diff.**
C1.R1	0.14	0.32	0.18	0.18	0.69	0.55	0.55	0.11	−0.03	0.03	0.23	0.09	0.09
C1.R2	0.17	0.33	0.16	0.16	0.71	0.54	0.54	0.11	−0.06	0.06	0.24	0.07	0.07
C1.R3	0.16	0.32	0.16	0.16	0.70	0.54	0.54	0.11	−0.05	0.05	0.24	0.08	0.08
C2.R1	0.14	0.82	0.68	0.68	1.0	0.86	0.86	0.27	0.13	0.13	0.33	0.19	0.19
C2.R2	0.14	0.68	0.54	0.54	0.86	0.72	0.72	0.22	0.08	0.08	0.28	0.14	0.14
K1.R1	0.24	2.6	2.36	2.36	1.1	0.86	0.86	0.85	0.61	0.61	0.35	0.11	0.11
K1.R2	0.61	3.0	2.39	2.39	1.2	0.59	0.59	0.96	0.35	0.35	0.39	−0.22	0.22
F1.R1	0.58	1.1	0.52	0.52	1.4	0.82	0.82	0.38	−0.20	0.20	0.47	−0.11	0.11
F1.R2	0.12	1.6	1.48	1.48	2.0	1.88	1.88	0.55	0.43	0.43	0.67	0.55	0.55
Q1.R1	0.15	0.76	0.61	0.61	1.3	1.15	1.15	0.26	0.11	0.11	0.44	0.29	0.29
Q1.R2	0.21	0.89	0.68	0.68	1.5	1.29	1.29	0.30	0.09	0.09	0.52	0.31	0.31
F2.R1	0.70	1.4	0.70	0.70	0.60	−0.10	0.10	0.68	−0.02	0.02	0.30	−0.4	0.4
F2.R2	0.29	1.4	1.11	1.11	0.59	0.30	0.30	0.66	0.37	0.37	0.29	0.0	0.0
K2.R1	0.14	1.8	1.66	1.66	1.4	1.26	1.26	0.83	0.69	0.69	0.64	0.50	0.50
K2.R2	0.05	1.8	1.75	1.75	1.4	1.35	1.35	0.82	0.77	0.77	0.63	0.58	0.58
**MAE**			**1.0**			**0.85**			**0.27**			**0.24**	
